# Gender Matters: the Need for a New Approach to Psychopathology

**DOI:** 10.1192/j.eurpsy.2022.2224

**Published:** 2022-09-01

**Authors:** R.J. Van Der Gaag, P. Van Wijngaarden-Cremers

**Affiliations:** 1 Stradina University, Psychosomatics And Psychotherapy, Riga, Latvia; 2 Dimence, Child & Adolescent, Deventer, Netherlands

**Keywords:** Epigenetic’s, Social Determinants, gender, classification

## Abstract

**Introduction:**

Treatment of mental problems is based on classification categories. Yet most patients display far more complex problems than discribed in those categories. In child psychiatry boys are overrepresented whereas in adults women are in the majority when it comes to mental health problems. This raises the question whether gender and diversity shouldn’t be taken more into account in order to come to better classifications and understanding of developemental psychopathology

**Objectives:**

To look into the influences of gender, genetics, stress, child rearing and social determinants on the development of psychopathology

**Methods:**

A literature search was performed with genetics, gender, stress and social determinants as keywords was in order to question the specificity and validity of current categories of psychopathology.

**Results:**

The search yielded 26 articles. Interestingly this supports the hypothesis that the focus on phenotypical classifications is misleading and that gender plays an important role in the expression of endophenotypes (psychophysiological and neuropsychological). Moreover in many cases gender is not taken in to account enough in studies and that gender biased conclusions (when the reseach has included more men than women for different reasons) are extrapolated to easily to the other sexe, assumoing that the outcomes are universal

**Conclusions:**

The categoral approach to psychopathology has stimulated research in a very productive fashion. Yet now we should think beyond categories in mental health and have the courage to adapt our clincial practice to endophenotypes taking into account the permanent interaction between individual and enviironment. Which implies a more gender specific approach to (psycho)pathology

**Disclosure:**

No significant relationships.

